# Species-specific content of thiamin (vitamin B_1_) in phytoplankton and the transfer to copepods

**DOI:** 10.1093/plankt/fbaa015

**Published:** 2020-04-27

**Authors:** Emil Fridolfsson, Elin Lindehoff, Catherine Legrand, Samuel Hylander

**Affiliations:** Centre for Ecology and Evolution in Microbial Model Systems - EEMiS, Department of Biology and Environmental Science, Linnaeus University, Pedalstråket 13, SE-39231 Kalmar, Sweden

**Keywords:** B-vitamins, zooplankton, trace element, microbial food web, primary producers

## Abstract

Thiamin (vitamin B_1_) is primarily produced by bacteria and phytoplankton in aquatic food webs and transferred by ingestion to higher trophic levels. However, much remains unknown regarding production, content and transfer of this water-soluble, essential micronutrient. Hence, the thiamin content of six phytoplankton species from different taxa was investigated, along with the effect of thiamin amendment on thiamin content. Furthermore, thiamin transfer to copepods was estimated in feeding experiments. Prey type, not phytoplankton thiamin content *per se*, was the most important factor for the transfer of thiamin, as it was lowest from filamentous Cyanophyceae and highest from more easily ingested prey like *Dunaliella tertiolecta* and *Rhodomonas salina*. Cyanophyceae had the highest thiamin content of the investigated species, eightfold higher than the lowest. Phytoplankton varied in thiamin content related to the supply of thiamin, where thiamin addition enabled higher thiamin content in some species, while copepod thiamin content was less variable. In all, thiamin transfer is not only dependent on the prey thiamin content, but also the edibility and/or digestibility is of importance. Thiamin is essential for all organisms, and this study constitutes an important building block to understanding the dynamics and transfer of thiamin in the aquatic food web.

## INTRODUCTION

Thiamin, previously called thiamine or aneurine (Combs, 2012), serves as a co-factor for enzymes involved in e.g. carbon metabolism and is thereby essential in all organisms and most cells (Jurgenson *et al.,* 2009; Manzetti *et al.,* 2014; Kraft and Angert, 2017). Still, the understanding on how thiamin is transferred in the ecosystem, from producers to consumers, is limited. Thiamin in aquatic systems is predominantly produced by bacteria, phytoplankton and fungi and is then transferred throughout the food web, to zooplankton, planktivorous and piscivorous fishes and birds (Webb *et al.,* 2007; Sañudo-Wilhelmy *et al.,* 2014). Thiamin is a water-soluble vitamin, implying that storage of the compound is limited and organisms that cannot produce thiamin *de novo*, so-called thiamin auxotrophs, require a constant supply (Jurgenson *et al.,* 2009). Thiamin synthesis consists of the production of the precursors, hydroxyethylthiazole (HET) and hydroxymethylpyrimidine (HMP), which are ultimately coupled to form thiamin (Settembre *et al.,* 2003).

Dissolved thiamin concentrations are very low in aquatic environments, around picomolar levels for most of the studied areas and have been shown to limit coastal phytoplankton communities (Gobler *et al.,* 2007; Koch *et al.,* 2012; Sañudo-Wilhelmy *et al.,* 2012). Furthermore, there seems to be an active transfer between the pools of dissolved and particulate thiamin in the world’s ocean (Suffridge *et al.,* 2018). The ability to produce thiamin is not present in all bacteria and phytoplankton, and the auxotrophy level in different phytoplankton phyla varies greatly. On average, the auxotrophy level is ~25% for all the investigated phytoplankton, where in some phyla, 86% of the species are thiamin auxotrophs, while some phyla are non-auxotrophic (prototrophic) (Carlucci and Bowes, 1970a, b; Croft *et al.,* 2006; Tang *et al.,* 2010). Previously, ~30% of the investigated marine bacteria strains were found to be thiamin auxotrophs (Burkholder, 1961). Recently, bacterial thiamin auxotrophy was found to be more pronounced and displayed larger seasonal variation than previously assumed (Paerl *et al.,* 2018b). Most thiamin auxotrophs only lack one or more crucial part of the synthetic pathway, and not the complete thiamin synthesis pathway (Combs, 2012; Kraft and Angert, 2017). Recently, it was discovered that some phytoplankton could use related precursors, other than HET and HMP, to sustain their thiamin requirement (Gutowska *et al.,* 2017; Paerl *et al.,* 2018a).

In addition to a high variability among different phytoplankton phyla in thiamin auxotrophy, thiamin content is also variable among different phytoplankton species (Brown *et al.,* 1999; Sylvander *et al.,* 2013; Gutowska *et al.,* 2017; Fridolfsson *et al.,* 2018). In previous studies, filamentous Cyanophyceae like *Aphanizomenon flos-aquae*, *Nodularia spumigena* and *Dolichospermum* sp. have been found to have relatively high thiamin content, while members of other classes like Bacillariophyceae, Chlorophyceae, Dinophyceae, Prymnesiophyceae and Cryptophyceae had lower concentrations (Sylvander *et al.,* 2013; Gutowska *et al.,* 2017; Fridolfsson *et al.,* 2018). Also under natural conditions, thiamin content of seston (bacteria, phytoplankton, protists and detritus) displayed the highest thiamin concentrations when filamentous cyanobacteria made a significant contribution to the phytoplankton community (Fridolfsson *et al.,* 2019). Copepod thiamin content correlated with the thiamin content of differently sized seston; however, highest thiamin concentration in seston did not cause elevated copepod thiamin content, indicating a periodically disrupted transfer of thiamin in the food web or that levels are above the requirements (Fridolfsson *et al.,* 2019).

Episodically, fish and bird populations in the Baltic Sea and the Laurentian Great Lakes suffer from thiamin deficiency, resulting in higher offspring mortality in salmonids and paralysis and mass death in bird populations (Bengtsson *et al.,* 1999; Fitzsimons *et al.,* 1999; Balk *et al.,* 2009; Balk *et al.,* 2016). It has been suggested that for salmon (*Salmo salar*) in the Baltic Sea, thiamin deficiency may be related to an unbalanced diet, consisting of prey with a low thiamin per unit energy, especially small-sized sprat (*Sprattus sprattus*) (Keinänen *et al.,* 2012). In the Great Lakes, thiamin deficiency is related to the prey alewife (*Alosa pseudoharengus*), which is rich in thiaminase I, an enzyme that degrades thiamin (Honeyfield *et al.,* 2005). However, interspecific differences in thiamin deficiency syndromes and thiamin levels exist between salmonines in the Great Lakes (Futia and Rinchard, 2019). Recently, the flow of thiamin in the food web was modeled and the results show that the thiamin levels in planktivorous fish is lower when mesozooplankton biomass is low and the biomass of small-sized phytoplankton (picoplankton) is high and stable over time (Ejsmond *et al.,* 2019). Scenarios of constrained thiamin transfer in the system were characterized by high nutrient levels and high abundance of planktivorous fish or alternatively by conditions of high light attenuation and high planktivorous fish abundance (Ejsmond *et al.,* 2019).

Hence, knowledge on thiamin dynamics in the ecosystem is important in order to understand thiamin deficiency issues in top predators. Also, the importance of phytoplankton thiamin content and the transfer to higher trophic levels is poorly understood. As the ability to produce thiamin differs among taxa, we hypothesize that also thiamin content differs among phytoplankton species. Furthermore, we hypothesize that the transfer of thiamin to zooplankton is species dependent and related to selective feeding. To target this knowledge gap, we investigated the effect of thiamin supply on phytoplankton thiamin content. Furthermore, we conducted a feeding and thiamin transfer experiment with the dominant zooplankton species and phytoplankton from six common taxa in the Baltic Sea.

## METHOD

### Phytoplankton cultures

To investigate if thiamin content differs between classes, the phytoplankton species included in the present study cover different taxonomic classes. Cultures were obtained from Kalmar Algae Collection (KAC), curated by Linnaeus University, and from the Marine Research Centre at the Finnish Environment Institute (SYKE MRC)/Tvärminne Zoological Station algal and cyanobacterial culture collection. Non-axenic, mono-specific cultures of Cyanophyceae *Nodularia spumigena* (KAC 11) and *Aphanizomenon flos-aquae* (KAC 15), Bacillariophyceae *Skeletonema marinoi* (Skeletonema), Prymnesiophyceae *Prymnesium parvum* (KAC 39), Chlorophyceae *Dunaliella tertiolecta* (CCMP 1302) and Cryptophyceae *Rhodomonas salina* (KAC 30) were grown under controlled conditions. Cultures were grown at 16°C under a light:dark cycle (16:8 h; intensity ~100 μmol photons s^−1^ m^−1^) with gentle aeration to promote growth. In the present study, each phytoplankton was cultivated in two different growth media treatments, Thiamin (296 nM thiamin) with full f/2 medium (Guillard, 1975) and Control with f/2 medium with no thiamin (0 nM) added. New inoculations were started every third to fourth day for both Thiamin and Control treatments during 1 month. New inoculations were made by transferring a small volume (~1 mL) of the culture to fresh media according to the two treatments to minimize the amount of thiamin present in the Control cultures. Cultures were maintained for 3 months prior to the start of the feeding experiment.

### Feeding experiment

To assess the transfer of thiamin at the base of the food web, feeding experiments with copepods and phytoplankton grown in the two different treatments were performed. Copepods were collected at Linnaeus Microbial Observatory (LMO; N 56° 55.8540′, E 17° 3.6420′), as described in Legrand *et al.* (2015), by oblique hauls from 30-m depth to the surface with a plankton net (200 μm mesh size, Ø 50 cm) (HELCOM, 2017). Copepods were acclimatized to experimental conditions for 2 days prior to the start of the feeding experiments. Experiments were conducted in 10-L containers with four replicates per phytoplankton and treatment. Phytoplankton from the stock cultures was added to the containers to reach the nominal concentration of ~ 1100 μg C L^−1^, well over food saturation (Holeton *et al.,* 2009). Copepod concentration was determined in a stock solution from which all treatments received a volumetric addition resulting in a nominal concentration of 75 adult copepods L^−1^, and was assumed similar in all replicates. The volume in the containers was adjusted to 8 L with filtered (1 μm) seawater. Hence, bacteria were present during the feeding experiment. Experiments were conducted under dark conditions, to minimize phytoplankton growth, at 16°C and lasted for 8 days. Concentrations of phytoplankton were monitored daily and additions (from cultures with the different treatments) were made every other day at levels to maintain the nominal concentrations. Phytoplankton and copepods were sampled initially and after 8 days. Copepods were kept in filtered seawater for at least 4 h prior to sampling for thiamin, carbon and nitrogen content. This was performed in order to allow for emptying the guts, to limit the amount of thiamin, carbon and nitrogen from undigested phytoplankton still left in the guts. Phytoplankton samples were collected by filtration on Whatman GF/F filters (25 mm), while for copepods, 40 and 20 adult individuals were carefully picked using tweezers under a stereomicroscope (Olympus SX7) for thiamin and carbon/nitrogen content analysis, respectively. Samples for thiamin analyses were stored in Eppendorf tubes at −80°C until further analysis. Samples for carbon and nitrogen content were collected on precombusted (475°C, 3 h) GF/F filters and stored in H_2_O_2_-washed Eppendorf tubes at −20°C until further analysis.

### Analysis of thiamin, carbon and nitrogen content

Thiamin was analyzed according to Pinto *et al.* (2002) with slight modifications according to Sylvander *et al.* (2013) and Fridolfsson *et al.* (2018). Briefly, thawed samples were sonicated in 1–1.5 mL of 0.1-M HCl with a Vibra-Cell sonicator (amplitude 92 for phytoplankton or 40 for copepod samples, respectively) with 1-s pulses for 1.5 min on ice. Extracts were centrifuged at 16 900$\times$*g* at 10°C for 10 min and 700 μL of this supernatant was centrifuged once more under the same conditions. Next, 600 μL of the supernatant was mixed with 550 μL of MeOH, 300 μL of 1-M NaOH and 50 μL of freshly made 30 mM K_3_Fe(CN)_6_. Finally, the mix was filtered through a 0.45-μm PTFE/PP syringe filter. Standard solutions (1 μM) for the three types of thiamin, free thiamin (TF), thiamin monophosphate (TMP) and thiamin diphosphate (TDP) were prepared in 0.1-M HCl and aliquoted in a five-point standard series. Blanks were prepared by mixing 600 μL of 0.1-M HCl with the other chemicals. Standards and blanks were treated just as samples, excepting sonication and centrifugation.

Thiamin samples were analyzed using a Hitachi Chromaster HPLC system with a Purospher®Star NH_2_ LiChroCART® column (5 μm particle size, 4.6 mm[I.D.]$\times$250 mm), protected by a guard column (5 μm particle size, 4 mm[I.D.]$\times$4 mm), and a fluorescence detector (excitation wavelength 375 nm, emission wavelength 450 nm). Samples were kept in the autosampler at 4°C and the column oven kept 30°C. 100 μL was injected with a flowrate of 1 mL min^−1^ and the mobile phase consisted of MeOH: 0.1 M phosphate buffer (pH 7.4) (43:57). Chromatograms were integrated using the software OpenLab (Agilent Technologies), and baselines were drawn automatically and inspected manually. Three types of thiamin were analyzed, TF, TMP and TDP, and these values were summed up in order to get the total thiamin content (T_tot_).

Prior to analysis of particulate organic carbon and nitrogen content, filters were dried at 60°C for at least 24 h before being stored in a desiccator and content was analyzed in a Perkin Elmer CHNS/O Analyzer 2400 Series II.

### Phytoplankton and zooplankton counting and growth rate

Phytoplankton abundance in the experimental containers was analyzed daily to ensure that the nominal concentrations were maintained, using a Sedgewick-Rafter counting chamber in an inverted microscope (Olympus CKX41). Counting unit was 100 μm of filaments for filamentous Cyanophyceae and individual cells for the remaining species, according to standard techniques (Olenina *et al.,* 2006). Phytoplankton growth rate was evaluated using 24-well plates and measuring optical density at 750 nm (OD750) daily with a plate reader (Floustar Omega, BMG Labtech). Growth rate was assessed in a separate growth experiment, performed in the 24-well plates (2 mL in well) using the same treatments used in the feeding experiment. Phytoplankton growth rate was calculated using the following equation, growth rate = ln(OD_b_/OD_a_)/*t*_b_ − *t*_a_, in which OD_b_ and OD_a_ are the OD750 values at respective time, *t*_b_ or *t*_a_. Zooplankton abundance was analyzed using a stereomicroscope (Olympus SX7).

### Data handling and statistical analyses

Statistical analyses were performed and graphics were created with R version 3.6.0 (R Core Team, 2019). Results from two-way ANOVA’s are presented as *F* values and *P* values and the results from the subsequent *post hoc* test (Tukey) (Hothorn *et al.,* 2008) are presented as *t* values. Thiamin content data in the present study are presented as two measurements. Carbon-specific thiamin content, calculated by dividing thiamin per sample by the carbon content per sample, and thiamin per individual copepod (T_tot_ ind^−1^) or counting unit (T_tot_ unit^−1^), calculated by dividing thiamin per sample by the number of individuals sampled or counting units. Unless stated otherwise, thiamin content refers to carbon-specific total thiamin content (T_tot_). Thiamin ratio was calculated by dividing the total thiamin content in the copepods by the total thiamin content in the phytoplankton prey, according to Hairston and Hairston (1993) and Fridolfsson *et al.* (2019). A thiamin ratio >1 implies that the thiamin content in copepods is higher than in its phytoplankton prey, indicating that copepods can assimilate thiamin and store it, at least for some period of time. Importantly, the thiamin ratio calculated here does not consider retention efficiency or cellular demand of thiamin.

## RESULTS

### Thiamin content in phytoplankton

**Fig. 1:**
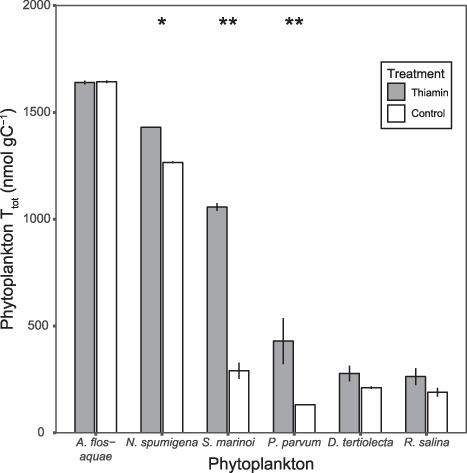
Total thiamin content in phytoplankton supplied with extra thiamin (Thiamin) or Control (no addition). Significance level indicated by stars, *P* < 0.01 (double asterisks), *P* < 0.05 (asterisks) and refer to statistical test between treatments within a phytoplankton species. Error bars illustrate standard deviation for replicates.

Total thiamin content (T_tot_) differed between the two treatments (Thiamin and Control) as well as between phytoplankton species, and there was an interaction effect between the two factors (*F*_(5,12)_ = 57.01, *P* < 0.05). Phytoplankton cultures supplied with extra thiamin (Thiamin) tended to have a higher T_tot_ than cultures not supplied with extra thiamin (Control) (Fig. 1), even if this difference between treatments was not present in all phytoplankton species. *A. flos-aquae*, *D. tertiolecta* and *R. salina* did not show any difference in T_tot_ between the two treatments. However, T_tot_ in *N. spumigena*, *S. marinoi* and *P. parvum* differed between the treatments, all being higher in the Thiamin treatment (Fig. 1; Supplementary Table S1).

Furthermore, T_tot_ was also different within treatments, between species, irrespective of thiamin amendment or not. Within the Thiamin cultures, *A. flos-aquae* had the highest T_tot_ (1640.3 ± 8.8), followed by *N. spumigena* (1430.3 ± 1.4), *S. marinoi* (1057 ± 18.0) and *P. parvum* (429.6 ± 107.8). Thiamin content did not differ significantly between *D. tertiolecta* and *R. salina* (278.0 ± 36.7 and 263.3 ± 39.7, respectively; Fig. 1; Supplementary Table S2). For the Control cultures, the trend was similar, even if the absolute values where generally lower for these cultures. Highest T_tot_ was found in *A. flos-aquae* (1643.6 ± 6.4), followed by *N. spumigena* (1266.0 ± 5.0). T_tot_ for *S. marinoi* without thiamin addition was lower than in the thiamin treatment (290.9 ± 38.0), which was close to the levels of *P. parvum* (131.3 ± 0.3), *D. tertiolecta* (211.3 ± 5.9) and *R. salina* (190 ± 20.4), (Fig. 1; Supplementary Table S2). T_tot_ unit^−1^ displayed a slightly different pattern from the carbon-specific phytoplankton thiamin content. T_tot_ unit^−1^ also differed between the two treatments as well as between phytoplankton species and an interaction effect was present between the two factors (*F*_(5,12)_ = 881.6, *P* < 0.05). Similar to carbon-specific thiamin content, T_tot_ unit^−1^was higher in the Thiamin cultures, for *N. spumigena*, *S. marinoi* and *P. parvum*. In contrast to carbon-specific thiamin content, T_tot_ unit^−1^ was also higher in the Thiamin cultures for *A. flos-aquae*, (Table I; Supplementary Table S1). For the two remaining species, T_tot_ unit^−1^ did not differ significantly between Thiamin and Control cultures, *D. tertiolecta* and *R. salina*, (Table I; Supplementary Table S1).

**Table I:** Average phytoplankton total thiamin content per counting unit, growth rate, carbon (C) and nitrogen (N) content and molar C:N ratio for different phytoplankton species in treatments with additional thiamin (Thiamin) and Control (no addition)

Phytoplankton	Treatment	T_tot_ (pmol (10^6^ units)^−1^)	Growth rate	Carbon content (pg C unit^−1^)	Nitrogen content (pg N unit^−1^)	Molar C:N
*A. flos-aquae*	Thiamin	**492.1 (±2.6)**	0.1 (±0.07)	**300 (±0.5)**	**56.5 (±0.1)**	6.2 (±0)
	Control	**392.8 (±1.5)**	0.08 (±0.04)	**239 (±8.6)**	**46.2 (±1.9)**	6 (±0)
*N. spumigena*	Thiamin	**973.1 (±1)**	0.06 (±0.02)	**680.3 (±3.9)**	**150.6 (±0.3)**	5.3 (±0)
	Control	**781.4 (±3.1)**	0.06 (±0.03)	**617.2 (±5.3)**	**137.1 (±0.3)**	5.2 (±0.1)
*S. marinoi*	Thiamin	**66.2 (±1.1)**	0.19 (±0.01)	62.7 (±0.3)	8.6 (±0.6)	8.5 (±0.6)
	Control	**18.5 (±2.4)**	0.18 (±0.03)	63.6 (±0.2)	9 (±0.1)	8.3 (±0.1)
*P. parvum*	Thiamin	**24.9 (±6.3)**	**0.22 (±0.02)**	58 (±2.6)	4.7 (±0.3)	**14.4 (±0.2)**
	Control	**6.9 (±0)**	**−0.02 (±0.03)**	52.2 (±1.3)	6.9 (±0.4)	**8.9 (±0.2)**
*D. tertiolecta*	Thiamin	12.9 (±1.7)	0.22 (±0.02)	46.3 (±1.4)	5.1 (±0.5)	10.6 (±0.8)
	Control	9.9 (±0.3)	0.22 (±0.01)	47.1 (±0.5)	5.6 (±0.5)	9.9 (±0.8)
*R. salina*	Thiamin	13.1 (±2)	0.11 (±0.01)	**56.4 (±7.5)**	**8.5 (±0.6)**	7.7 (±0.6)
	Control	18.1 (±1.9)	0.05 (±0.03)	**98 (±4.4)**	**18 (±1.4)**	6.4 (±0.3)

*Values in parenthesis are standard deviation. Values in bold represent significant (*P *< 0.05) differences between treatments.*

### Thiamin content in copepods

**Fig. 2:**
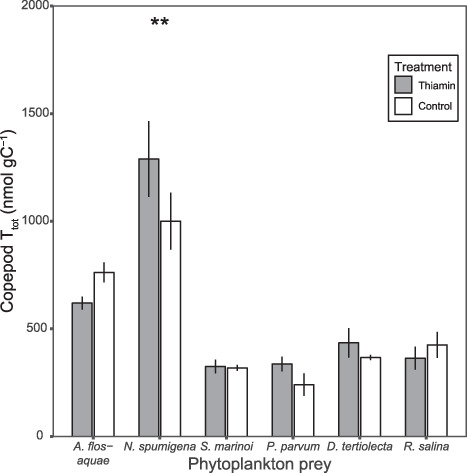
Total thiamin content in copepods fed phytoplankton supplied with extra thiamin (Thiamin) or Control (no addition). Significance level indicated by stars, *P* < 0.01 (double asterisks), *P* < 0.05 (asterisks) and refer to statistical test between treatments within a phytoplankton species. Error bars illustrate standard deviation for replicates.

Copepod T_tot_ differed between the two treatments as well as between phytoplankton prey species, and an interaction effect between the two factors was present (*F*_(5,33)_ = 6.865, *P* < 0.05). T_tot_ in copepods fed with phytoplankton that had received additional thiamin (Thiamin) or Control treatment (no addition) only differed significantly for one phytoplankton prey, *N. spumigena* (Fig. 2). Copepods fed *N. spumigena* in the Thiamin treatment had higher T_tot_ than copepods fed *N. spumigena* from the control. For copepods fed with the other phytoplankton species, no significant differences were present between the treatments within the prey species, (Fig. 2; Supplementary Table S3).

Copepods feeding on different phytoplankton species displayed a difference in T_tot_, both for copepods fed with Thiamin and Control phytoplankton cultures (Fig. 2; Supplementary Tables S3 and S4). For copepods fed Thiamin cultures, highest T_tot_ was found in copepods fed *N. spumigena* (1289.3 ± 175.9), followed by copepods fed *A. flos-aquae* (619.5 ± 30.1). Copepods fed the remaining phytoplankton species had lower T_tot_ (*S. marinoi* (324.6 ± 32.1), *P. parvum* (336.6 ± 34.6), *D. tertiolecta* (434.7 ± 68.2) and *R. salina* (363.7 ± 53.7)), and the levels did not differ significantly depending on prey species between these four (Fig. 2; Supplementary Table S4). The pattern was similar for copepods fed control cultures, with highest T_tot_ in copepods fed *N. spumigena* (1000.5 ± 132.9) and *A. flos-aquae* (762.2 ± 46.3). T_tot_ in copepods provided with the other four prey species was lower (*S. marinoi* (317.9 ± 13.9), *P. parvum* (240.1 ± 52.3), *D. tertiolecta* (366.4 ± 11.9) and *R. salina* (425.1 ± 60.7)), and did not differ between the phytoplankton cultures in the control treatment (Fig. 2; Supplementary Table S4). Copepod T_tot_ differed significantly compared to *in situ* copepod T_tot_ (348 ± 9, data not shown) for filamentous Cyanophyceae treatments, being higher after the feeding experiment, but not the other phytoplankton species. Moreover, T_tot_ ind^−1^ did not differ significantly between the two treatments, but there was a significant difference between phytoplankton prey species, and an interaction effect between the two factors was present (*F*_(5,33)_ = 3.625, *P* < 0.05). However, minor differences were present in T_tot_ ind^−1^ (Table II; Supplementary Tables S3 and S4).

**Table II:** Average copepod total thiamin content per individual, ingestion and mortality rate, carbon (C) and nitrogen (N) content and molar C:N ratio for the different phytoplankton prey species in treatments with additional thiamin (Thiamin) and Control (no addition)

Phytoplankton prey	Treatment	T_tot_ (pmol ind^−1^)	Ingestion rate (μg C ind^−1^ day^−1^)	Mortality rate (% copepods day^−1^)	Carbon content (μg C ind^−1^)	Nitrogen content (μg N ind^−1^)	Molar C:N
*A. flos-aquae*	Thiamin	0.7 (±0)	0.9 (0–2)	3.3 (±0.3)	1.1 (±0.2)	0.2 (±0.1)	5.5 (±1.9)
	Control	0.8 (±0)	1 (0.3–1.6)	15.5 (±6)	1 (±0.2)	0.3 (±0.2)	5.5 (±2.1)
*N. spumigena*	Thiamin	0.7 (±0.1)	0.8 (0.5–1)	20.4 (±10.4)	0.5 (±0.1)	0.1 (±0.1)	6.7 (±3.3)
	Control	0.6 (±0.1)	0.3 (0.1–0.5)	25.5 (±16.5)	0.6 (±0.3)	0.2 (±0.1)	4.7 (±0.9)
*S. marinoi*	Thiamin	0.7 (±0.1)	3.1 (0.7–4.8)	3.3 (±0.3)	2 (±0.4)	0.6 (±0.2)	4.4 (±1.1)
	Control	0.7 (±0)	3.7 (1.5–6)	0 (±0)	2.3 (±0.7)	0.6 (±0.1)	4.7 (±0.4)
*P. parvum*	Thiamin	1.1 (±0.1)	0.1 (0.1–0.2)	4.7 (±0.5)	3.4 (±0.6)	0.6 (±0.3)	7.4 (±3.5)
	Control	0.9 (±0.2)	0.1 (0–0.1)	16.6 (±6.9)	3.7 (±0.2)	0.8 (±0)	5.2 (±0.4)
*D. tertiolecta*	Thiamin	0.8 (±0.1)	0.8 (0–2.6)	3.3 (±0.3)	1.9 (±0.4)	0.5 (±0.1)	4.7 (±1.5)
	Control	0.8 (±0)	1.1 (0.4–2.9)	3.3 (±0.3)	2.1 (±0.3)	0.6 (±0.1)	4 (±0.4)
*R. salina*	Thiamin	0.8 (±0.1)	2.2 (0.1–6.7)	15.5 (±6)	2.3 (±0.3)	0.5 (±0.1)	5.6 (±1)
	Control	1 (±0.1)	3.3 (0.6–6.1)	8.1 (±1.6)	2.4 (±0.2)	0.7 (±0.1)	4.4 (±0.9)

*Values in parenthesis are minimum and maximum value for ingestion rate and standard deviation for the other variables*

### Phytoplankton growth rate and elemental composition

Parallel to the feeding experiment, growth rate was investigated to examine the effect of thiamin addition. Growth rates differed between the two treatments as well as between phytoplankton species, and an interaction effect was present between the two factors (*F*_(5,36)_ = 17, *P* < 0.05). Within phytoplankton species, growth rate only differed significantly between treatments for one species, *P. parvum* (*t* = −10.659, *P* < 0.05) (Table I; Supplementary Table S1), with higher growth rate in Thiamin treatment. For the other phytoplankton species, there was no significant differences between the Thiamin treatment and the Control, (Table I; Supplementary Table S1). Within the two treatments, growth rates differed between phytoplankton species (Table I; Supplementary Table S5). There were also significant differences of phytoplankton carbon and nitrogen content between Thiamin and Control, as well as between phytoplankton species, and there was an interaction effect between the two factors for both carbon (*F*_(5,12)_ = 147.6, *P* < 0.05) and nitrogen (*F*_(5,12)_ = 161.07, *P* < 0.05), (Table I; Supplementary Tables S1 and S5). Carbon and nitrogen content was higher in the two Cyanophyceae, *A. flos-aquae* and *N. spumigena* compared to the other phytoplankton strains, and the trend was similar for both the elemental compounds (Table I; Supplementary Table S5). In contrast, the C:N ratio was lower in the two Cyanophyceae and *R. salina* compared to the other strains, and in addition, the C:N ratio differed significantly between the treatments and phytoplankton species, with an interaction effect present (*F*_(5,12)_ = 24.57, *P* < 0.05), (Table I; Supplementary Table S5).

### Copepod elemental composition, ingestion, mortality rate and community composition

**Fig. 3:**
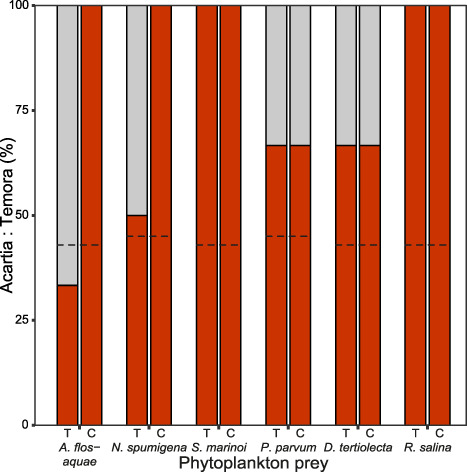
Copepod community composition displayed as *Acartia*: *Temora* ratio in treatments with extra thiamin (Thiamin, T) or Control, C (no addition) for the different phytoplankton prey at endpoint. Dashed line represent *Acartia*: *Temora* ratio the start of the feeding experiment. Proportion of *Acartia* is in red while *Temora* is in gray.

Carbon content in copepods differed significantly, depending on which phytoplankton prey they were fed (*F*_(5,40)_ = 66.453, *P* < 0.05), but not comparing the two treatments (*F*_(1,40)_ = 1.156, *P* = 0.289) (Table II). Copepod nitrogen content also differed significantly between treatments (*F*_(1,40)_ = 4.934, *P* < 0.05) and different phytoplankton prey (*F*_(5,40)_ = 21.235, *P* < 0.05) (Table II), with no significant interaction effect between the two factors. C:N ratio in copepods did not differ significantly between treatments (*F*_(1,40)_ = 3.571, *P* = 0.07) or phytoplankton prey (*F*_(5,40)_ = 1.428, *P* = 0.235) (Table II). Ingestion rates (μg C copepod^−1^ day^−1^) for copepods was estimated, and there was a significant difference in ingestion rates between phytoplankton prey (*F*_(5,47)_ = 8.268, *P* < 0.05), however not between treatments (*F*_(1,47)_ = 1.331, *P* = 0.254), (Table II). As the feeding experiment was conducted under dark conditions, no growth of the phytoplankton was assumed. Mortality rates (% copepods day^−1^) showed significant differences between treatments and phytoplankton prey, and there was an interaction effect present between the two factors (*F*_(5,84)_ = 6.270, *P* < 0.05). For Thiamin treatments, mortality rates were higher for copepods fed *N. spumigena* and *R. salina* (Table II). The copepod community consisted of *Acartia* sp. and *Temora longicornis*. At the start of the experiment, the *Acartia* sp.: *T. longicornis* ratio was on average 44:56 and after 8 days, it had shifted with a relative increase of *Acartia* sp. over *T. longicornis* in all replicates, except in the copepods that were fed *A. flos-aquae* from the Thiamin treatment, where *Acartia* sp.: *T. longicornis* ratio was 33:67 (Fig. 3).

### Relationship of thiamin between copepods and prey

**Fig. 4:**
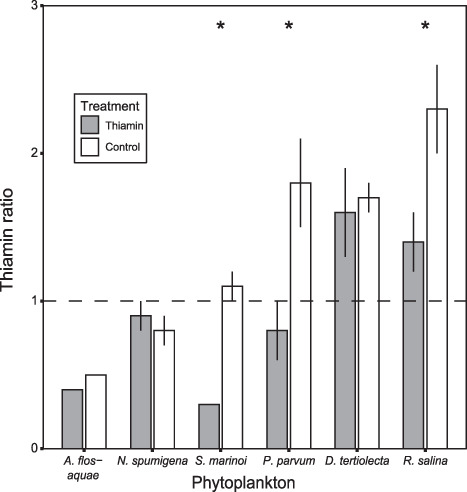
Thiamin ratio in treatments with extra thiamin (Thiamin) or Control (no addition) for the different phytoplankton prey. Dashed line illustrate a thiamin ratio of 1, which implies that the thiamin content in copepods the same as its phytoplankton prey. Significance level indicated by stars, *P* < 0.01 (double asterisks), *P* < 0.05 (asterisks) and refer to statistical test between treatments within a phytoplankton species. Error bars illustrate standard deviation for replicates.

To illustrate the transfer of thiamin from phytoplankton to copepods, thiamin ratio was calculated by dividing copepod T_tot_ with the phytoplankton T_tot_. Thiamin ratio differed between treatments and phytoplankton prey, and there was an interaction effect between the two factors (*F*_(5,78)_ = 20.48, *P* < 0.05). In Thiamin treatment, thiamin ratio was lowest in *A. flos-aquae* and *S. marinoi*, followed by *N. spumigena* and *P. parvum* and highest in *D. tertiolecta* and *R. salina*, and the trend was similar in the control treatment (Fig. 4; Supplementary Table S4). In some of the different phytoplankton prey species, there were significant differences in thiamin ratio between treatments, e.g. *S. marinoi*, *P. parvum* and *R. salina*, being higher in the Control treatments. Thiamin ratio did not differ significantly within *A. flos-aquae*, *N. spumigena* or *D. tertiolecta,* (Fig. 4; Supplementary Table S3).

## DISCUSSION

This study aims to increase our understanding of the dynamics of thiamin transfer from producers to consumers in the food web, a poorly understood topic that has bearing on thiamin deficiencies in higher trophic levels. As thiamin is produced mainly by bacteria and phytoplankton in the aquatic environments (Carlucci and Bowes, 1970a, b), and zooplankton is a crucial part of the food web, connecting the microbial producers and higher trophic levels, we focused our study on the transfer of thiamin from phytoplankton to copepods.

Addition of thiamin in the growth media induced higher total thiamin content (T_tot_) in some of the phytoplankton species, both when considering carbon-specific content and thiamin per counting unit, showing a plasticity of thiamin content related to the supply of thiamin (Fig. 1; Table I). Thiamin content of phytoplankton has been shown to be affected by abiotic stressors like salinity, temperature and light levels (Sylvander *et al.,* 2013). Recently, the thiamin content of a phytoplankton community was shown to be reduced by the presence of copepods, presumably due to selective feeding (Fridolfsson *et al.,* 2018), further illustrating that phytoplankton thiamin content could be affected by both abiotic and biotic factors. Even though thiamin addition to the cultures increased the thiamin content for some of the phytoplankton species, it was not true for all. These differences could be due to several factors, e.g. the maximum potential to accumulate thiamin, different uptake rates or the cellular demand for thiamin comparing different phytoplankton species. Future research should focus on which factors that affect the thiamin content, as interspecific differences in thiamin content is an important aspect to consider when investigating the transfer of thiamin. Of the investigated phytoplankton species, the two Cyanophyceae displayed the highest T_tot_, regardless of thiamin amendment (Fig. 1). Filamentous Cyanophyceae have previously been identified to contain high levels of thiamin previously (Sylvander *et al.,* 2013; Fridolfsson *et al.,* 2018), and the levels in the present study are similar to the prior findings. The remainder of the investigated phytoplankton had lower T_tot_, and all were comparable with members of the same class and other phytoplankton species in the few previous reports of phytoplankton thiamin content that are available in the literature (Sylvander *et al.,* 2013; Gutowska *et al.,* 2017; Fridolfsson *et al.,* 2018).

Copepods displayed different T_tot_ depending on which phytoplankton species they were fed, and whether the phytoplankton had received additional thiamin or not (Fig. 2). Even if the levels of thiamin were highest in copepods fed Cyanophyceae compared to copepods fed the other prey species, the thiamin ratio was lowest with these prey items (Fig. 4). Copepods feeding on the two Cyanophyceae had a thiamin ratio <1, indicating that the transfer of thiamin is low with filamentous Cyanophyceae (Fig. 4) and did not reach the potential optimal transfer. Also, the ingestion rates were also the lowest for the two filamentous Cyanophyceae (Table II). However, the thiamin ratio was >1 when *D. tertiolecta* or *R. salina* was supplied, irrespective of thiamin amendment treatment. Even if T_tot_ of the copepods fed with these two phytoplankton species was not the highest in this study, the transfer of thiamin was highest (Fig. 4). Previously, *R. salina* has been deemed a good food item for copepods, supporting growth and development (Dahl *et al.,* 2009), which is consistent with our study where *R. salina* enabled an efficient transfer of thiamin to *Acartia*. In the present study, copepods were only fed monostrain cultures and could not feed selectively; nevertheless, when fed a combination of filamentous Cyanophyceae and *R. salina*, the transfer of thiamin was also reduced when Cyanophyceae filaments were present (Fridolfsson *et al.,* 2018). Furthermore, in natural conditions, when filamentous Cyanophyceae constitute the majority of a phytoplankton community, the thiamin ratio was also low compared to when Cyanophyceae was not as present (Fridolfsson *et al.,* 2019). Generally, filamentous Cyanophyceae occurrence is coupled to negative effects for zooplankton, mostly related to egg production (Engström-Öst *et al.,* 2002). These negative effects are suggested to be due to the production of non-ribosomal peptides, e.g. nodularin (Mazur-Marzec *et al.,* 2016), poor composition of fatty acids (Ahlgren *et al.,* 1992) and difficulties handling the filaments (Gliwicz and Siedlar, 1980). Still, some zooplankton can tolerate filamentous Cyanophyceae and can reproduce and develop successfully in the presence of filamentous Cyanophyceae (Hogfors *et al.,* 2014). In the present study, the ingestion rate and carbon content was lower and the mortality rate was elevated when copepods were exposed to filamentous Cyanophyceae (Table II), showing that the copepods are having difficulties feeding on the filaments and that ingestion comes with negative effects on the survival (Walve and Larsson, 1999; Engström-Öst *et al.,* 2011).

Thiamin amendment to the phytoplankton cultures only caused elevated thiamin levels in copepods fed *N. spumigena*, compared to copepods fed the Control cultures (Fig. 2). For the other food items, copepod total thiamin content was similar, irrespective of thiamin addition. The fact that thiamin addition did not induce elevated thiamin levels in copepods fed with most of the phytoplankton species could indicate that there is a limit to how much thiamin copepods can accumulate. Furthermore, since there was a difference in copepod T_tot_ related to the different foods, thiamin content is influenced by not only the thiamin content of the food, but also to the food type. One factor that also should be considered is the copepod community composition in the experiment, as thiamin content has been shown to differ between copepod species in natural environments (Fridolfsson *et al.,* 2019). During the course of the experiment, *Acartia* sp. increased relatively to *T. longicornis* in most treatments (Fig. 3). This shift in community composition was equal within the different phytoplankton prey, except for the two filamentous Cyanophyceae, so some caution should be taken when comparing copepod thiamin content in the Thiamin and Control treatment in copepods fed filamentous Cyanophyceae. There were more pronounced differences in T_tot_ among the phytoplankton taxa than in the copepods, which further indicates that copepods have limited ability to accumulate thiamin (Fig. 1; Fig. 2). In the food web, copepods could therefore align the amount of thiamin being transferred to higher trophic, by reducing some of the variation in thiamin content observed among phytoplankton. Large variation in phytoplankton thiamin is not reflected in zooplankton, but prey type and thiamin concentration affect thiamin transfer even if copepod thiamin levels are much more similar over time and seasons (Fridolfsson *et al.,* 2019). Prey type as well as the associated edibility/digestibility and not the thiamin content *per se* was the most important for the transfer of thiamin from phytoplankton to copepods. B vitamins have been shown to limit the growth of natural phytoplankton communities (Gobler *et al.,* 2007; Koch *et al.,* 2012), however, to our knowledge, limitation in copepods has not been demonstrated and future studies should aim to investigate the potential of thiamin limitation of copepods.

In the present study, thiamin auxotrophy is unknown for two of the species (*A. flos-aquae* and *N. spumigena*), two are potential thiamin prototrophs (*S. marinoi* and *D. tertiolecta*) and two are thiamin auxotrophs (*P. parvum and R. salina*) (Croft *et al.,* 2006; Tang *et al.,* 2010). It is not known if filamentous Cyanophyceae can produce thiamin (Sañudo-Wilhelmy *et al.,* 2014), but to our knowledge, no thiamin auxotrophy has been reported in Cyanophyceae. For the two potential thiamin prototrophs, auxotrophy has not been investigated for the specific species, but members of the same genus have been determined to not require external thiamin in previous studies (Provasoli and Carlucci, 1974; Croft *et al.,* 2006; Tang *et al.,* 2010). However, *D. tertiolecta* can exude thiamin at nM levels (Carini *et al.,* 2014). Thiamin addition affected the growth differently among species, even if the phytoplankton cultures in the present study were not axenic. If one considers the filamentous Cyanophyceae as thiamin prototrophs, the growth rate for all of non-auxotrophs was similar between the two treatments (Table I). For *P. parvum*, an auxotroph, addition of thiamin increased the growth rate, with no growth in the Control treatment. The trend was similar for *R. salina*, also a thiamin auxotroph, however this species did not differ significantly between treatments. This suggests that the bacteria did not provide enough thiamin for their needs or that the interactions between the phytoplankton and bacteria were sub-optimal. In addition, the total thiamin content of the two auxotrophs tended to be higher in the Thiamin cultures than in the Control cultures. All experiments were non-axenic with potential contribution of bacteria in the analyses. Bacteria are tightly associated with phyto- and zooplankton communities and future studies would be needed to disentangle the individual contributions of bacteria and phytoplankton to the overall thiamin concentrations in seston. Attached and free-living bacteria may contribute to the total thiamin content as well as to the supply of dissolved thiamin (Cruz-López *et al.,* 2018), but these dynamics are beyond the scope of the present study and require further studies.

Thiamin amendment also increased the T_tot_ for some of the prototrophs, meaning that even if the species can produce thiamin *de novo*, extra-cellular thiamin can boost the cellular thiamin content. Some bacteria and phytoplankton have been found to be able to regulate their thiamin synthesis, depending on the extracellular and intracellular thiamin levels, using riboswitches (Rodionov *et al.,* 2002; Croft *et al.,* 2007). The capacity to boost the thiamin content, or accumulate, as well as the ability to regulate the thiamin synthesis depending on the surrounding concentrations could have a large effect on the thiamin dynamics in the aquatic food web. Thiamin content was similar for *S. marinoi*, *D. tertiolecta*, *P. parvum* and *R. salina*, the two latter being known thiamin auxotrophs. This suggests that thiamin auxotrophs might be an equally good source of thiamin as prototrophs for higher trophic levels, when dissolved thiamin is available. This finding illustrates that using thiamin auxotrophy to estimate thiamin supply could be a simplification, in contrast to what was recently suggested (Ruess and Müller-Navarra, 2019). This is further demonstrated by the finding that copepod T_tot_ did not differ significantly between these four phytoplankton preys, with similar thiamin levels in copepods irrespective of prey thiamin content (Fig. 2). Furthermore, when copepods are able to feed on the prey efficiently, copepods seem to be able to accumulate thiamin, i.e. a thiamin ratio >1 (Fig. 4). In contrast, when the food is more problematic to ingest and digest, copepod thiamin content was lower than its prey, i.e. a thiamin ratio <1. Even if a prey item has been ingested, it can still be difficult to digest, for instance cyanobacteria and chlorophytes are difficult to digest, whilst diatoms and cryptophytes are more easily digested (Porter, 1973). Hence, the results of the present study indicate that copepods can boost their thiamin content, at least for some period of time (i.e. thiamin ratio >1). However, field studies have always found thiamin ratios <1 (Fridolfsson *et al.,* 2019). Carbon-specific thiamin content was highest in copepods fed filamentous Cyanophyceae but these copepods were smaller (low carbon content) than copepods fed the other phytoplankton species (Table II). As a result, thiamin per individual copepod was similar between thiamin treatments and when comparing the different phytoplankton prey (Table II). Consequently, thiamin content was similar per individual for small and large copepods and this could indicate that the capacity to store thiamin is independent of size, alternatively that copepods can maintain thiamin homeostasis per individual but not per carbon content. Homeostasis have been observed for other nutrients and compounds in zooplankton (Andersen and Hessen, 1991; Brett *et al.,* 2006), but to our knowledge, prior to the present study, thiamin homeostasis has not been studied before in zooplankton.

## CONCLUSION

Thiamin originates from the lower trophic levels, such as bacteria and phytoplankton, which include both thiamin prototrophs and auxotrophs. Our study provides new insights into the transfer of thiamin from phytoplankton to copepods, as well as into the information on species-specific thiamin content of phytoplankton species, under both thiamin replete and deplete conditions. As shown in the present study, T_tot_ differs between phytoplankton species and can be boosted by thiamin addition to the cultures. Furthermore, the transfer of thiamin from phytoplankton to copepods, as indicated by the thiamin ratio, was lower for filamentous Cyanophyceae than the other phytoplankton taxa, probably due to difficulties in the handling, ingesting and/or digesting the filaments. In addition, copepod T_tot_ was higher than the thiamin content of some of the phytoplankton prey species demonstrating that copepods can boost their thiamin reserves above food item concentrations. In all, this suggests that transfer of thiamin is not only dependent on the thiamin content of the phytoplankton prey, but also highlights the importance of how easily copepods can access the thiamin, e.g. ingestion and digestion of the phytoplankton prey. Little is known about the transfer of thiamin from one trophic level to the next, and thus, this study constitutes an important building block in our knowledge on the dynamics of production and transfer of this micronutrient to higher trophic levels of the food web.

## Supplementary Material

Fridolfsson_et_al_JPR_Suppl_Revision_JPR-2019-146_R1_fbaa015

## References

[ref1] Ahlgren G., Gustafsson I.-B. and Boberg M. (1992) Fatty acid content and chemical composition of freshwater microalgae. J. Phycol., 28, 37–50.

[ref2] Andersen T. and Hessen D. O. (1991) Carbon, nitrogen, and phosphorus content of freshwater zooplankton. Limnol. Oceanogr., 36, 807–814.

[ref3] Balk L., Hägerroth P. Å., Gustavsson H., Sigg L., Åkerman G., Ruiz Munoz Y., Honeyfield D. C., Tjärnlund U. et al. (2016) Widespread episodic thiamine deficiency in northern hemisphere wildlife. Sci. Rep., 6, 38821. 27958327 10.1038/srep38821 PMC5153840

[ref4] Balk L., Hägerroth P. Å., Åkerman G., Hanson M., Tjärnlund U., Hansson T., Hallgrimsson G. T., Zebühr Y. et al. (2009) Wild birds of declining European species are dying from a thiamine deficiency syndrome. PNAS, 106, 12001–12006. 19597145 10.1073/pnas.0902903106 PMC2715476

[ref5] Bengtsson B.-E., Hill C., Bergman Å., Brandt I., Johansson N., Magnhagen C., Södergren A. and Thulin J. (1999) Reproductive disturbances in Baltic fish: a synopsis of the FiRe project. Ambio, 28, 2–8.

[ref6] Brett M., Müller-Navarra D. C., Ballantyne A. P., Ravet J. L. and Goldman C. R. (2006) Daphnia fatty acid composition reflects that of their diet. Limnol. Oceanogr., 51, 2428–2437.

[ref7] Brown M. R., Mular M., Miller I., Farmer C. and Trenerry C. (1999) The vitamin content of microalgae used in aquaculture. J Appl Phycol, 11, 247–255.

[ref8] Burkholder P. R. (1961) Some nutritional relationships among microbes of sea sediments and waters In Oppenheimer C. H. (ed.), Symposium on Marine Microbiology, Charles C Thomas, Springfield, IL, USA, pp. 133–150.

[ref9] Carini P., Campbell E. O., Morre J., Sanudo-Wilhelmy S. A., Thrash J. C., Bennett S. E., Temperton B., Begley T. et al. (2014) Discovery of a SAR11 growth requirement for thiamin's pyrimidine precursor and its distribution in the Sargasso Sea. ISME J., 8, 1727–1738. 24781899 10.1038/ismej.2014.61 PMC4817611

[ref10] Carlucci A. F. and Bowes P. M. (1970a) Production of vitamin B_12_, thiamine and biotin by phytoplankton. J. Phycol., 6, 351–357.

[ref11] Carlucci A. F. and Bowes P. M. (1970b) Vitamin production and utilization by phytoplankton in mixed culture. J. Phycol., 6, 393–400.

[ref12] Combs G. F. (2012) The Vitamins, Academic Press, San Diego, CA, USA.

[ref13] Croft M. T., Moulin M., Webb M. E. and Smith A. G. (2007) Thiamine biosynthesis in algae is regulated by riboswitches. PNAS, 104, 20770–20775. 18093957 10.1073/pnas.0705786105 PMC2410077

[ref14] Croft M. T., Warren M. J. and Smith A. G. (2006) Algae need their vitamins. Eukaryot. Cell, 5, 1175–1183. 16896203 10.1128/EC.00097-06 PMC1539151

[ref15] Cruz-López R., Maske H., Yarimizu K. and Holland N. A. (2018) The B-vitamin mutualism between the Dinoflagellate *Lingulodinium polyedrum* and the bacterium *Dinoroseobacter shibae*. Front. Mar. Sci., 5, 274.

[ref16] Dahl U., Lind C. R., Gorokhova E., Eklund B. and Breitholtz M. (2009) Food quality effects on copepod growth and development: implications for bioassays in ecotoxicological testing. Ecotoxicol. Environ. Saf., 72, 351–357. 18514311 10.1016/j.ecoenv.2008.04.008

[ref17] Ejsmond M. J., Blackburn N., Fridolfsson E., Haecky P., Andersson A., Casini M., Belgrano A. and Hylander S. (2019) Modeling vitamin B_1_ transfer to consumers in the aquatic food web. Sci. Rep., 9, 10045. 31296876 10.1038/s41598-019-46422-2 PMC6624374

[ref18] Engström-Öst J., Hogfors H., El-Shehawy R., De Stasio B., Vehmaa A. and Gorokhova E. (2011) Toxin-producing cyanobacterium *Nodularia spumigena*, potential competitors and grazers: testing mechanisms of reciprocal interactions. Aquat. Microb. Ecol., 62, 39–48.

[ref19] Engström-Öst J., Viitasalo M., Jónasdóttir S., Repka S., Sivonen K., Koski M. and Schmidt K. (2002) Calanoid copepods feed and produce eggs in the presence of toxic cyanobacteria *Nodularia spumigena*. Limnol. Oceanogr., 47, 878–885.

[ref20] Fitzsimons J. D., Brown S. B., Honeyfield D. C. and Hnath J. G. (1999) A review of early mortality syndrome (EMS) in Great Lakes Salmonids: relationship with thiamine deficiency. Ambio, 28, 9–15.

[ref21] Fridolfsson E., Bunse C., Legrand C., Lindehoff E., Majaneva S. and Hylander S. (2019) Seasonal variation and species-specific concentrations of the essential vitamin B_1_ (thiamin) in zooplankton and seston. Mar. Biol., 166, 70.

[ref22] Fridolfsson E., Lindehoff E., Legrand C. and Hylander S. (2018) Thiamin (vitamin B_1_) content in phytoplankton and zooplankton in the presence of filamentous cyanobacteria. Limnol. Oceanogr., 63, 2423–2435.

[ref23] Futia M. H. and Rinchard J. (2019) Evaluation of adult and offspring thiamine deficiency in salmonine species from Lake Ontario. J. Great Lakes Res., 45, 811–820.

[ref24] Gliwicz Z. M. and Siedlar E. (1980) Food size limitation and algae interfering with food collection in *Daphnia*. Arch Hydrobiol, 88, 155–177.

[ref25] Gobler C. J., Norman C., Panzeca C., Taylor G. T. and Sañudo-Wilhelmy S. A. (2007) Effect of B-vitamins (B_1_, B_12_) and inorganic nutrients on algal bloom dynamics in a coastal ecosystem. Aquat. Microb. Ecol., 49, 181–194.

[ref26] Guillard R. R. L. (1975) Culture of phytoplankton for feeding marine invertebrates In Smith W. L. and Chanley M. H. (eds.), Culture of Marine Invertebrates, Plenum Press, New York, USA, pp. 26–60.

[ref27] Gutowska M. A., Shome B., Sudek S., McRose D. L., Hamilton M., Giovannoni S. J., Begley T. P. and Worden A. Z. (2017) Globally important Haptophyte algae use exogenous pyrimidine compounds more efficiently than Thiamin. MBio, 8(5):e01459-17. 10.1128/mBio.01459-17 PMC5635689 29018119

[ref28] Hairston N. G. J. and Hairston N. G. S. (1993) Cause-effect relationships in energy flow, trophic structure, and interspecific interactions. Am. Nat., 142, 379–411.

[ref29] HELCOM (2017) Manual for Marine Monitoring in the COMBINE Programme, HELCOM Combine, Helsinki. http://www.helcom.fi/action-areas/monitoring-and-assessment/manuals-and-guidelines/combine-manual

[ref30] Hogfors H., Motwani N. H., Hajdu S., El-Shehawy R., Holmborn T., Vehmaa A., Engström-Öst J., Brutemark A. et al. (2014) Bloom-forming cyanobacteria support copepod reproduction and development in the Baltic Sea. PLoS One, 9, e112692. 25409500 10.1371/journal.pone.0112692 PMC4237358

[ref31] Holeton C., Lindell K., Holmborn T., Hogfors H. and Gorokhova E. (2009) Decreased astaxanthin at high feeding rates in the calanoid copepod *Acartia bifilosa*. J. Plankton Res., 31, 661–668.

[ref32] Honeyfield D. C., Hinterkopf J. P., Fitzsimons J. D., Tillitt D. E., Zajicek J. L. and Brown S. B. (2005) Development of thiamine deficiencies and early mortality syndrome in Lake trout by feeding experimental and feral fish diets containing Thiaminase. J. Aquat. Anim. Health, 17, 4–12.

[ref33] Hothorn T., Bretz F. and Westfall P. (2008) Simultaneous inference in general parametric models. Biom. J., 50, 346–363. 18481363 10.1002/bimj.200810425

[ref34] Jurgenson C. T., Begley T. P. and Ealick S. E. (2009) The structural and biochemical foundations of thiamin biosynthesis. Annu. Rev. Biochem., 78, 569–603. 19348578 10.1146/annurev.biochem.78.072407.102340 PMC6078420

[ref35] Keinänen M., Uddström A., Mikkonen J., Casini M., Pönni J., Myllylä T., Aro E. and Vuorinen P. J. (2012) The thiamine deficiency syndrome M74, a reproductive disorder of Atlantic salmon (*Salmo salar*) feeding in the Baltic Sea, is related to the fat and thiamine content of prey fish. ICES J Mar Sci, 69, 516–528.

[ref36] Koch F., Hattenrath-Lehmann T. K., Goleski J. A., Sañudo-Wilhelmy S., Fisher N. S. and Gobler C. J. (2012) Vitamin B_1_ and B_12_ uptake and cycling by plankton communities in coastal ecosystems. Front. Microbiol., 3, 363. 23091470 10.3389/fmicb.2012.00363 PMC3469840

[ref37] Kraft C. E. and Angert E. R. (2017) Competition for vitamin B_1_ (thiamin) structures numerous ecological interactions. Q. Rev. Biol., 92, 151–168. 29562121 10.1086/692168

[ref38] Legrand C., Fridolfsson E., Bertos-Fortis M., Lindehoff E., Larsson P., Pinhassi J. and Andersson A. (2015) Interannual variability of phyto-bacterioplankton biomass and production in coastal and offshore waters of the Baltic Sea. Ambio, 44, 427–438. 26022325 10.1007/s13280-015-0662-8 PMC4447688

[ref39] Manzetti S., Zhang J. and Spoel D. van der (2014) Thiamin function, metabolism, uptake, and transport. Biochemistry, 53, 821–835. 24460461 10.1021/bi401618y

[ref40] Mazur-Marzec H., Bertos-Fortis M., Torunska-Sitarz A., Fidor A. and Legrand C. (2016) Chemical and genetic diversity of *Nodularia spumigena* from the Baltic Sea. Mar. Drugs, 14, 209. 10.3390/md14110209 PMC5128752 27834904

[ref41] Olenina I., Hajdu S., Edler L., Andersson A., Wasmund N., Busch S., Göbel J., Gromisz S. et al. (2006) Biovolumes and size-classes of phytoplankton in the Baltic Sea. HELCOM Balt Sea Environ Proc, 106, 144.

[ref42] Paerl R. W., Bertrand E. M., Rowland E., Schatt P., Mehiri M., Niehaus T. D., Hanson A. D., Riemann L. et al. (2018a) Carboxythiazole is a key microbial nutrient currency and critical component of thiamin biosynthesis. Sci. Rep., 8, 5940. 29654239 10.1038/s41598-018-24321-2 PMC5899164

[ref43] Paerl R. W., Sundh J., Tan D., Svenningsen S. L., Hylander S., Andersson A. F., Pinhassi J. and Riemann L. (2018b) Prevalent reliance of bacterioplankton on exogenous vitamin B1 and precursor availability. PNAS, 115, E10447–E10456. 30322929 10.1073/pnas.1806425115 PMC6217396

[ref44] Pinto E., Pedersen M., Snoeijs P., Van Nieuwerburgh L. and Colepicolo P. (2002) Simultaneous detection of thiamine and its phosphate esters from microalgae by HPLC. Biochem. Biophys. Res. Commun., 291, 344–348. 11846410 10.1006/bbrc.2002.6438

[ref45] Porter K. G. (1973) Selective grazing and differential digestion of algae by zooplankton. Nature, 244, 179–180.

[ref46] Provasoli L. and Carlucci A. F. (1974) Vitamins and growth regulators In Stewart W. D. P. (ed.), Algal Physiology and Biochemistry, Blackwell Scientific Publications, London, pp. 741–787.

[ref47] R Core Team (2019) R: A Language and Environment for Statistical Computing, R Foundation for Statistical Computing, Vienna, Austria.

[ref48] Rodionov D. A., Vitreschak A. G., Mironov A. A. and Gelfand M. S. (2002) Comparative genomics of thiamin biosynthesis in procaryotes. New genes and regulatory mechanisms. J. Biol. Chem., 277, 48949–48959. 12376536 10.1074/jbc.M208965200

[ref49] Ruess L. and Müller-Navarra D. C. (2019) Essential biomolecules in food webs. Front. Ecol. Evol., 7, 269.

[ref50] Sañudo-Wilhelmy S. A., Cutter L. S., Durazo R., Smail E. A., Gómez-Consarnau L., Webb E. A., Prokopenko M. G., Berelson W. M. et al. (2012) Multiple B-vitamin depletion in large areas of the coastal ocean. PNAS, 109, 14041–14045. 22826241 10.1073/pnas.1208755109 PMC3435217

[ref51] Sañudo-Wilhelmy S. A., Gómez-Consarnau L., Suffridge C. and Webb E. A. (2014) The role of B vitamins in marine biogeochemistry. Ann. Rev. Mar. Sci., 6, 339–367. 10.1146/annurev-marine-120710-100912 24050603

[ref52] Settembre E., Begley T. P. and Ealick S. E. (2003) Structural biology of enzymes of the thiamin biosynthesis pathway. Curr. Opin. Struct. Biol., 13, 739–747. 14675553 10.1016/j.sbi.2003.10.006

[ref53] Suffridge C. P., Gómez-Consarnau L., Monteverde D. R., Cutter L., Arístegui J., Alvarez-Salgado X. A., Gasol J. M. and Sañudo-Wilhelmy S. A. (2018) B-vitamins and their congeners as potential drivers of microbial community composition in an oligotrophic marine ecosystem. Eur. J. Vasc. Endovasc. Surg., 123, 2890–2907.

[ref54] Sylvander P., Häubner N. and Snoeijs P. (2013) The thiamine content of phytoplankton cells is affected by abiotic stress and growth rate. Microb. Ecol., 65, 566–577. 23263236 10.1007/s00248-012-0156-1

[ref55] Tang Y. Z., Koch F. and Gobler C. J. (2010) Most harmful algal bloom species are vitamin B_1_ and B_12_ auxotrophs. PNAS, 107, 20756–20761. 21068377 10.1073/pnas.1009566107 PMC2996436

[ref56] Walve J. and Larsson U. (1999) Carbon, nitrogen and phosphorus stoichiometry of crustacean zooplankton in the Baltic Sea: implications for nutrient recycling. J. Plankton Res., 21, 2309–2321.

[ref57] Webb M. E., Marquet A., Mendel R. R., Rebeille F. and Smith A. G. (2007) Elucidating biosynthetic pathways for vitamins and cofactors. Nat. Prod. Rep., 24, 988–1008. 17898894 10.1039/b703105j

