# Supplementary material for: Species-specific content of thiamin (vitamin B1) in phytoplankton and the transfer to copepods
Source: J Plankton Res. 2020 Apr 27;42(3):274–85. doi: 10.1093/plankt/fbaa015 (PMC7252500; doi:10.1093/plankt/fbaa015)
Supplement: Fridolfsson_et_al_JPR_Suppl_Revision_JPR-2019-146_R1_fbaa015 [file fridolfsson_et_al_jpr_suppl_revision_jpr-2019-146_r1_fbaa015.docx]

# Supplemental material

Species-specific content of thiamin (vitamin B_1_) in phytoplankton and the transfer to copepods

Emil Fridolfsson^1^*, Elin Lindehoff^1^, Catherine Legrand^1^, Samuel Hylander^1^

^1^Linnaeus University, Centre for Ecology and Evolution in Microbial model Systems - EEMiS, SE-39182 Kalmar, Sweden.

ORCID:
Emil Fridolfsson; 0000-0003-4871-7441
Elin Lindehoff; 0000-0002-1149-6852
Catherine Legrand; 0000-0001-7155-3604
Samuel Hylander; 0000-0002-3740-5998

*** Correspondence:**Emil Fridolfsson
emil.fridolfsson@lnu.se
+4673 - 41 65 878

Keywords: B-vitamins; zooplankton; trace element; microbial food web; primary producers

## Supplemental tables

**Table S1.** Results from Tukey's post-hoc test for differences in phytoplankton total thiamin content, normalized either by carbon content or by 10^6^ counting units, growth rate, carbon and nitrogen content and molar C:N ratio, between treatments within phytoplankton species. Significance (Sig.) level indicated by stars, p<0.001 (***), p<0.01 (**), p<0.05 (*).

| Phytoplankton | Treatment | | T_tot_ (nmol (gC)^-1^) | | T_tot_ (pmol (10^6^ units)^-1^) | | Growth rate | | Carbon content (pg C unit^-1^) | | Nitrogen content (pg N unit^-1^) | | Molar C:N | |
| --- | --- | --- | --- | --- | --- | --- | --- | --- | --- | --- | --- | --- | --- | --- |
|  |  |  | t value | Sig. level | t value | Sig. level | t value | Sig. level | t value | Sig. level | t value | Sig. level | t value | Sig. level |
| *A. flos-aquae* | Thiamin | Control | -0.086 |  | 39.213 | ***** | 1.247 |  | 17.818 | ***** | 15.445 | ***** | 0.380 |  |
| *N. spumigena* | Thiamin | Control | 4.375 | *** | 75.699 | ***** | -0.227 |  | 18.434 | ***** | 20.219 | ***** | 0.046 |  |
| *S. marinoi* | Thiamin | Control | 20.400 | ***** | 18.858 | ***** | 0.567 |  | -0.270 |  | -0.582 |  | 0.634 |  |
| *P. parvum* | Thiamin | Control | 7.942 | ***** | 7.141 | ***** | 10.659 | ***** | 1.708 |  | -3.229 |  | 12.960 | ***** |
| *D. tertiolecta* | Thiamin | Control | 1.777 |  | 1.153 |  | 0.113 |  | -0.230 |  | -0.713 |  | 1.750 |  |
| *R. salina* | Thiamin | Control | 1.951 |  | -1.958 |  | 2.608 |  | -13.249 | ***** | -13.990 | ***** | 2.049 |  |

**Table S2.** Results from Tukey's post-hoc test for differences in phytoplankton total thiamin content, normalized either by carbon content or by 10^6^ counting units (100 µm filaments for Cyanophyceae and individual cells for the other species), within treatments between phytoplankton species. Significance (Sig.) level indicated by stars, p<0.001 (***), p<0.01 (**), p<0.05 (*).

| Treatment | Phytoplankton | | T_tot_ (nmol (gC)^-1^) | | T_tot_ (pmol (10^6^ units)^-1^) | |
| --- | --- | --- | --- | --- | --- | --- |
|  |  |  | t value | Sig. level | t value | Sig. level |
| Thiamin | *A. flos-aquae* | *N. spumigena* | -5.594 | ** | 189.967 | *** |
|  |  | *S. marinoi* | -15.535 | *** | -168.198 | *** |
|  |  | *P. parvum* | -32.242 | *** | -184.515 | *** |
|  |  | *D. tertiolecta* | -36.278 | *** | -189.283 | *** |
|  |  | *R. salina* | -36.671 | *** | -189.169 | *** |
|  | *N. spumigena* | *S. marinoi* | -9.941 | *** | -358.165 | *** |
|  |  | *P. parvum* | -26.649 | *** | -374.481 | *** |
|  |  | *D. tertiolecta* | -30.684 | *** | -379.250 | *** |
|  |  | *R. salina* | -31.077 | *** | -379.136 | *** |
|  | *S. marinoi* | *P. parvum* | -16.708 | *** | -16.317 | *** |
|  |  | *D. tertiolecta* | -20.743 | *** | -21.085 | *** |
|  |  | *R. salina* | -21.136 | *** | -20.971 | *** |
|  | *P. parvum* | *D. tertiolecta* | -4.035 | * | -4.768 | * |
|  |  | *R. salina* | -4.428 | * | -4.655 | * |
|  | *D. tertiolecta* | *R. salina* | -0.393 |  | 0.113 |  |
| Control | *A. flos-aquae* | *N. spumigena* | -10.055 | *** | 153.481 | *** |
|  |  | *S. marinoi* | -36.021 | *** | -147.843 | *** |
|  |  | *P. parvum* | -40.271 | *** | -152.442 | *** |
|  |  | *D. tertiolecta* | -38.141 | *** | -151.223 | *** |
|  |  | *R. salina* | -38.708 | *** | -147.998 | *** |
|  | *N. spumigena* | *S. marinoi* | -25.966 | *** | -301.323 | *** |
|  |  | *P. parvum* | -30.216 | *** | -305.923 | *** |
|  |  | *D. tertiolecta* | -28.086 | *** | -304.704 | *** |
|  |  | *R. salina* | -28.653 | *** | -301.479 | *** |
|  | *S. marinoi* | *P. parvum* | -4.250 | * | -4.600 | * |
|  |  | *D. tertiolecta* | -2.120 |  | -3.381 |  |
|  |  | *R. salina* | -2.687 |  | -0.156 |  |
|  | *P. parvum* | *D. tertiolecta* | 2.130 |  | 1.219 |  |
|  |  | *R. salina* | 1.563 |  | 4.444 | * |
|  | *D. tertiolecta* | *R. salina* | -0.567 |  | 3.225 |  |

**Table S3.** Results from Tukey's post-hoc test for differences in copepod total thiamin content, normalized either by carbon content or by individuals and thiamin ratio, between treatments within phytoplankton preys. Significance (Sig.) level indicated by stars, p<0.001 (***), p<0.01 (**), p<0.05 (*).

| Phytoplankton prey | Treatment | | T_tot_ (nmol (gC)^-1^) | | T_tot_ (pmol ind.^-1^) | | Thiamin ratio | | |
| --- | --- | --- | --- | --- | --- | --- | --- | --- | --- |
|  |  |  | t value | Sig. level | t value | Sig. level | | t value | Sig. level |
| *A. flos-aquae* | Thiamin | Control | -2.388 |  | -1.285 |  | | -0.784 |  |
| *N. spumigena* | Thiamin | Control | 5.217 | ***** | 0.328 |  | | 1.093 |  |
| *S. marinoi* | Thiamin | Control | 0.122 |  | -1.045 |  | | -7.818 | *** |
| *P. parvum* | Thiamin | Control | 1.614 |  | 3.026 |  | | -9.279 | *** |
| *D. tertiolecta* | Thiamin | Control | 1.142 |  | 0.516 |  | | -1.433 |  |
| *R. salina* | Thiamin | Control | -1.110 |  | -2.462 |  | | -8.389 | *** |

**Table S4.** Results from Tukey's post-hoc test for differences in copepod total thiamin content, normalized either by carbon content or by individuals and thiamin ratio, within treatments between phytoplankton preys. Significance (Sig.) level indicated by stars, p<0.001 (***), p<0.01 (**), p<0.05 (*).

| Treatment | Phytoplankton prey | | T_tot_ (nmol (gC)^-1^) | | T_tot_ (pmol ind.^-1^) | | Thiamin ratio | |
| --- | --- | --- | --- | --- | --- | --- | --- | --- |
|  |  |  | t value | Sig. level | t value | Sig. level | t value | Sig. level |
| Thiamin | *A. flos-aquae* | *N. spumigena* | 11.203 | *** | -0.152 |  | 4.769 | *** |
|  |  | *S. marinoi* | -4.932 | ** | -0.245 |  | -0.642 |  |
|  |  | *P. parvum* | -4.425 | ** | 5.433 | *** | 3.674 | * |
|  |  | *D. tertiolecta* | -3.091 |  | 1.640 |  | 10.921 | *** |
|  |  | *R. salina* | -4.279 | ** | 2.027 |  | 9.283 | *** |
|  | *N. spumigena* | *S. marinoi* | -17.428 | *** | -0.100 |  | -5.844 | *** |
|  |  | *P. parvum* | -15.934 | *** | 5.960 | *** | -0.841 |  |
|  |  | *D. tertiolecta* | -15.439 | *** | 1.936 |  | 6.645 | *** |
|  |  | *R. salina* | -16.722 | *** | 2.353 |  | 4.876 | *** |
|  | *S. marinoi* | *P. parvum* | 0.201 |  | 6.053 | *** | 4.570 | ** |
|  |  | *D. tertiolecta* | 1.988 |  | 2.036 |  | 12.490 | *** |
|  |  | *R. salina* | 0.706 |  | 2.453 |  | 10.720 | *** |
|  | *P. parvum* | *D. tertiolecta* | 1.640 |  | -4.168 | ** | 6.993 | *** |
|  |  | *R. salina* | 0.452 |  | -3.782 | * | 5.355 | *** |
|  | *D. tertiolecta* | *R. salina* | -1.283 |  | 0.418 |  | -1.770 |  |
| Control | *A. flos-aquae* | *N. spumigena* | 4.305 | ** | -1.880 |  | 3.211 |  |
|  |  | *S. marinoi* | -8.028 | *** | -0.608 |  | 6.277 | *** |
|  |  | *P. parvum* | -9.433 | *** | 1.617 |  | 13.419 | *** |
|  |  | *D. tertiolecta* | -6.621 | *** | -0.161 |  | 11.570 | *** |
|  |  | *R. salina* | -6.091 | *** | 3.263 |  | 17.569 | *** |
|  | *N. spumigena* | *S. marinoi* | -12.332 | *** | 1.273 |  | 3.066 |  |
|  |  | *P. parvum* | -13.737 | *** | 3.497 |  | 10.207 | *** |
|  |  | *D. tertiolecta* | -10.606 | *** | 1.580 |  | 8.597 | *** |
|  |  | *R. salina* | -10.395 | *** | 5.144 | *** | 14.358 | *** |
|  | *S. marinoi* | *P. parvum* | -1.405 |  | 2.225 |  | 7.141 | *** |
|  |  | *D. tertiolecta* | 0.811 |  | 0.402 |  | 5.759 | *** |
|  |  | *R. salina* | 1.937 |  | 3.871 | * | 11.292 | *** |
|  | *P. parvum* | *D. tertiolecta* | 2.112 |  | -1.658 |  | -0.853 |  |
|  |  | *R. salina* | 3.342 |  | 1.647 |  | 4.150 | ** |
|  | *D. tertiolecta* | *R. salina* | 0.982 |  | 3.182 |  | 4.695 | *** |

**Table S5.** Results from Tukey's post-hoc test for differences in phytoplankton growth rate, carbon and nitrogen content and molar C:N ratio within treatments between phytoplankton preys. Significance (Sig.) level indicated by stars, p<0.001 (***), p<0.01 (**), p<0.05 (*).

| Treatment | Phytoplankton | | Growth rate | | Carbon content (pg C unit^-1^) | | | Nitrogen content (pg N unit^-1^) | | Molar C:N | | | |
| --- | --- | --- | --- | --- | --- | --- | --- | --- | --- | --- | --- | --- | --- |
|  |  |  | t value | Sig. level | t value | Sig. level | t value | | Sig. level | | t value | Sig. level |  |
| Thiamin | *A. flos-aquae* | *N. spumigena* | -1.928 |  | 111.103 | *** | 141.384 | | *** | | -2.184 |  |  |
|  |  | *S. marinoi* | 3.969 | *** | -69.326 | *** | -71.950 | | *** | | 5.506 | ** |  |
|  |  | *P. parvum* | 5.329 | ***** | -70.678 | *** | -77.781 | | *** | | 19.288 | *** |  |
|  |  | *D. tertiolecta* | 5.443 | ***** | -74.121 | *** | -77.215 | | *** | | 10.498 | *** |  |
|  |  | *R. salina* | 0.340 |  | -73.045 | *** | -72.855 | | *** | | 2.600 |  |  |
|  | *N. spumigena* | *S. marinoi* | 5.896 | ***** | -180.429 | *** | -213.334 | | *** | | 7.690 | *** |  |
|  |  | *P. parvum* | 7.257 | ***** | -181.781 | *** | -219.165 | | *** | | 21.472 | *** |  |
|  |  | *D. tertiolecta* | 7.370 | ***** | -185.224 | *** | -218.599 | | *** | | 12.682 | *** |  |
|  |  | *R. salina* | 2.268 |  | -184.148 | *** | -214.239 | | *** | | 4.783 | * |  |
|  | *S. marinoi* | *P. parvum* | 1.361 |  | -1.352 |  | -5.831 | | ** | | 13.782 | *** |  |
|  |  | *D. tertiolecta* | 1.474 |  | -4.795 | * | -5.265 | | ** | | 4.992 | * |  |
|  |  | *R. salina* | -3.628 | *** | -3.719 |  | -0.905 | |  | | -2.906 |  |  |
|  | *P. parvum* | *D. tertiolecta* | 0.113 |  | -3.442 |  | 0.566 | |  | | -8.790 | *** |  |
|  |  | *R. salina* | -4.989 | ***** | -2.367 |  | 4.926 | | * | | -16.688 | *** |  |
|  | *D. tertiolecta* | *R. salina* | -5.103 | ***** | 1.076 |  | 4.360 | | * | | -7.898 | *** |  |
| Control | *A. flos-aquae* | *N. spumigena* | -0.454 |  | 110.487 | *** | 136.611 | | *** | | -1.850 |  |  |
|  |  | *S. marinoi* | 4.649 | **** | -51.238 | *** | -55.923 | | *** | | 5.251 | ** |  |
|  |  | *P. parvum* | -4.082 | *** | -54.568 | *** | -59.106 | | *** | | 6.708 | *** |  |
|  |  | *D. tertiolecta* | 6.577 | ***** | -56.072 | *** | -61.057 | | *** | | 9.127 | *** |  |
|  |  | *R. salina* | -1.021 |  | -41.978 | *** | -43.419 | | *** | | 0.930 |  |  |
|  | *N. spumigena* | *S. marinoi* | 5.103 | ***** | -161.725 | *** | -192.533 | | *** | | 7.101 | *** |  |
|  |  | *P. parvum* | -3.628 | *** | -165.055 | *** | -195.717 | | *** | | 8.558 | *** |  |
|  |  | *D. tertiolecta* | 7.030 | ***** | -166.559 | *** | -197.667 | | *** | | 10.977 | *** |  |
|  |  | *R. salina* | -0.567 |  | -152.465 | *** | -180.030 | | *** | | 2.780 |  |  |
|  | *S. marinoi* | *P. parvum* | -8.731 | ***** | -3.330 |  | -3.184 | |  | | 1.457 |  |  |
|  |  | *D. tertiolecta* | 1.928 |  | -4.834 | * | -5.134 | | ** | | 3.876 |  |  |
|  |  | *R. salina* | -5.669 | ***** | 9.261 | *** | 12.503 | | *** | | -4.321 | * |  |
|  | *P. parvum* | *D. tertiolecta* | 10.659 | ***** | -1.504 |  | -1.950 | |  | | 2.419 |  |  |
|  |  | *R. salina* | 3.062 |  | 12.591 | *** | 15.687 | | *** | | -5.778 | ** |  |
|  | *D. tertiolecta* | *R. salina* | -7.597 | ***** | 14.095 | *** | 17.637 | | *** | | -8.197 | *** |  |
